# Supplementary material for: Trade-off between training and testing ratio in machine learning for medical image processing
Source: PeerJ Comput Sci. 2024 Sep 6;10:e2245. doi: 10.7717/peerj-cs.2245 (PMC11419616; doi:10.7717/peerj-cs.2245)
Supplement: Supplemental Information 2 [file peerj-cs-10-2245-s002.pdf]

PYTHON CODE FOR BRAIN TUMOR CLASSIFICATION INCLUDING DATA PREPROCESSING AND CROSS VALIDATION WITH DESCRIPTION

```
# Import necessary libraries

import pandas as pd

import numpy as np

import matplotlib.pyplot as plt

import seaborn as sns

from sklearn.model_selection import KFold, train_test_split, cross_val_score

from sklearn.preprocessing import StandardScaler

from sklearn.linear_model import LogisticRegression

from sklearn.ensemble import RandomForestClassifier

from sklearn.neighbors import KNeighborsClassifier

from sklearn.svm import SVC

from xgboost import XGBClassifier

from sklearn.metrics import accuracy_score, confusion_matrix

from sklearn.feature_selection import SelectKBest, chi2


# Data Preprocessing

# Load the dataset (modify the path according to your environment)

# Assuming 'data.csv' is your dataset file

df = pd.read_csv('path_to_your_dataset.csv')


# Initial exploration

print("First five rows of the dataset:")

print(df.head())
```

```
print("\nShape of the dataset:")
print(df.shape)

print("\nChecking for missing values:")
print(df.isnull().sum())

# Assuming 'label' is the target column
print("\nUnique labels in the dataset:")
print(df['label'].unique())

# Splitting data into features and target
X = df.drop('label', axis=1)
y = df['label']

# Handling missing values if any
X.fillna(X.mean(), inplace=True)

# Feature Scaling
scaler = StandardScaler()
X_scaled = scaler.fit_transform(X)

# Splitting data into training and testing sets
X_train, X_test, y_train, y_test = train_test_split(X_scaled, y, test_size=0.2, random_state=42)
```

```
# Models initialization
models = {
    'Logistic Regression': LogisticRegression(),
    'Random Forest': RandomForestClassifier(),
    'K-Nearest Neighbors': KNeighborsClassifier(),
    'Support Vector Classifier': SVC(),
    'XGBoost': XGBClassifier()
}

# Cross-Validation
kf = KFold(n_splits=10, shuffle=True, random_state=42)

for model_name, model in models.items():
    cv_results = cross_val_score(model, X_train, y_train, cv=kf, scoring='accuracy')
    print(f"{model_name} - Cross-Validation Accuracy: {cv_results.mean():.4f} ± {cv_results.std():.4f}")

# Model Evaluation on Test Set
for model_name, model in models.items():
    model.fit(X_train, y_train)
    y_pred = model.predict(X_test)
    accuracy = accuracy_score(y_test, y_pred)
    print(f"{model_name} - Test Accuracy: {accuracy:.4f}")
    print(f"Confusion Matrix:\n{confusion_matrix(y_test, y_pred)}\n")
```

Description of Code

Imports:

Import necessary libraries for data manipulation, visualization, and model training.

Data Preprocessing:

1. Load the dataset.
2. Display initial dataset information (first few rows, shape, missing values, unique labels).
3. Split data into features (X) and target (y).
4. Handle missing values by filling them with the mean of respective columns.
5. Scale the features using StandardScaler.
6. Split the scaled data into training and testing sets.

Model Initialization:

Initialize various classification models.

Cross-Validation:

Use KFold for 10-fold cross-validation.

Evaluate each model using cross-validation and print the average accuracy and standard deviation.

Model Evaluation on Test Set:

Train each model on the training set.

Predict and evaluate accuracy on the test set.

Display the confusion matrix for each model.
