# Supplementary material for: Trade-off between training and testing ratio in machine learning for medical image processing
Source: PeerJ Comput Sci. 2024 Sep 6;10:e2245. doi: 10.7717/peerj-cs.2245 (PMC11419616; doi:10.7717/peerj-cs.2245)
Supplement: Supplemental Information 4 [file peerj-cs-10-2245-s004.pdf]

MATLAB CODE FOR FEATURE EXTRACTION USING GLCM WITH DESCRIPTION

```
function [GLCM_stats] = GLCM_Features1(glcmin, pairs)

% GLCM_Features1 calculates features from the GLCM.

%

% INPUT:

% glcmin - A 3D matrix of GLCMs.

% pairs - (Unused) parameter for compatibility.

%

% OUTPUT:

% GLCM_stats - A vector of calculated GLCM features.


% Get the size of the GLCM

[size_glcm_1, size_glcm_2, size_glcm_3] = size(glcmin);


% Initialize variables

out = struct();

out.autoc = zeros(size_glcm_3, 1);

out.contr = zeros(size_glcm_3, 1);

out.corrm = zeros(size_glcm_3, 1);

out.corrp = zeros(size_glcm_3, 1);

out.cprom = zeros(size_glcm_3, 1);

out.cshad = zeros(size_glcm_3, 1);

out.dissi = zeros(size_glcm_3, 1);

out.energ = zeros(size_glcm_3, 1);

out.entro = zeros(size_glcm_3, 1);
```

```
out.homom = zeros(size_glcm_3, 1);

out.homop = zeros(size_glcm_3, 1);

out.maxpr = zeros(size_glcm_3, 1);

out.sosvh = zeros(size_glcm_3, 1);

out.savgh = zeros(size_glcm_3, 1);

out.svarh = zeros(size_glcm_3, 1);

out.senth = zeros(size_glcm_3, 1);

out.dvarh = zeros(size_glcm_3, 1);

out.denth = zeros(size_glcm_3, 1);

out.inf1h = zeros(size_glcm_3, 1);

out.inf2h = zeros(size_glcm_3, 1);

out.indnc = zeros(size_glcm_3, 1);

out.idmnc = zeros(size_glcm_3, 1);


% Process each GLCM slice

for k = 1:size_glcm_3

    glcm = glcmin(:, :, k);

    glcm_sum = sum(glcm(:));

    glcm = glcm / glcm_sum;


    % Indices for GLCM

    [i, j] = meshgrid(1:size_glcm_1, 1:size_glcm_2);


    % Autocorrelation

    out.autoc(k) = sum(sum((i .* j) .* glcm));
```

```
% Contrast
out.contr(k) = sum(sum((abs(i - j) .^ 2) .* glcm));


% Correlation
u_i = sum(i .* sum(glcm, 2));
u_j = sum(j .* sum(glcm, 1));
s_i = sum(sum(((i - u_i) .^ 2) .* glcm));
s_j = sum(sum(((j - u_j) .^ 2) .* glcm));
out.corrm(k) = sum(sum((i - u_i) .* (j - u_j) .* glcm)) / sqrt(s_i * s_j);


% Cluster Prominence and Cluster Shade
out.cprom(k) = sum(sum(((i + j - u_i - u_j) .^ 4) .* glcm));
out.cshad(k) = sum(sum(((i + j - u_i - u_j) .^ 3) .* glcm));


% Dissimilarity
out.dissi(k) = sum(sum(abs(i - j) .* glcm));


% Energy
out.energ(k) = sum(sum(glcm .^ 2));


% Entropy
out.entro(k) = -sum(sum(glcm .* log(glcm + eps)));


% Homogeneity
```

```
out.homom(k) = sum(sum(glcm ./ (1 + abs(i - j))));

% Maximum Probability
out.maxpr(k) = max(glcm(:));

% Sum of Squares: Variance
out.sosvh(k) = sum(sum((i - u_i) .^ 2 .* glcm));

% Sum Average
out.savgh(k) = sum(sum((i + j) .* glcm)) / 2;

% Sum Variance
out.svarh(k) = var(i(:) .* glcm(:));

% Sum Entropy
out.senth(k) = -sum(sum(glcm .* log(glcm + eps)));

% Difference Variance
out.dvarh(k) = var(abs(i - j) .* glcm(:));

% Difference Entropy
out.denth(k) = -sum(sum(abs(i - j) .* glcm .* log(glcm + eps)));

% Information Measures of Correlation
HXY = out.entro(k);
```

```
    HX = -sum(sum(sum(glcm, 2) .* log(sum(glcm, 2) + eps)));

    HY = -sum(sum(sum(glcm, 1) .* log(sum(glcm, 1) + eps)));

    HXY1 = -sum(sum(glcm .* log(sum(glcm, 2) * sum(glcm, 1) + eps)));

    HXY2 = -sum(sum((sum(glcm, 2) * sum(glcm, 1)) .* log(sum(glcm, 2) * sum(glcm, 1) + eps)));

    out.inf1h(k) = (HXY - HXY1) / max(HX, HY);

    out.inf2h(k) = sqrt(1 - exp(-2 * (HXY2 - HXY)));


    % Inverse Difference Normalized (INN)

    out.indnc(k) = sum(sum(glcm ./ (1 + (i - j) .^ 2 / size_glcm_1 ^ 2)));


    % Inverse Difference Moment Normalized (IDN)

    out.idmnc(k) = sum(sum(glcm ./ (1 + abs(i - j) / size_glcm_1)));

end


% Consolidate the GLCM features into a single vector

GLCM_stats = [mean(out.autoc), mean(out.contr), mean(out.corrm), mean(out.corrp), mean(out.cprom), mean(out.cshad), ...

            mean(out.dissi), mean(out.energ), mean(out.entro), mean(out.homom), mean(out.homop), mean(out.maxpr), ...

            mean(out.sosvh), mean(out.savgh), mean(out.svarh), mean(out.senth), mean(out.dvarh), mean(out.denth), ...

            mean(out.inf1h), mean(out.inf2h), mean(out.indnc), mean(out.idmnc)];

end
```

Description

1. Initialization: All features are initialized as zero matrices of appropriate size.

2. Normalization: Each GLCM slice is normalized by dividing by its sum.
3. Vectorized Calculations: Features are calculated using matrix operations to improve efficiency.
4. Feature Aggregation: The final features are averaged across all GLCM slices.

Usage

To use this function, ensure your input glcmin is a 3D matrix of GLCMs and call the function as follows:

GLCM_stats = GLCM_Features1(glcmin, pairs);

Replace glcmin and pairs with your actual data. The pairs parameter is currently unused but retained for compatibility.
